# Supplementary material for: Beliefs, Practices and Health Care Seeking Behavior of Parents Regarding Fever in Children
Source: Medicina (Kaunas). 2019 Jul 22;55(7):398. doi: 10.3390/medicina55070398 (PMC6681325; doi:10.3390/medicina55070398)
Supplement: Supplementary file 1 [file medicina-55-00398-s001.zip › supplementary_files/informed_consent.pdf]

Informed consent form for the parents / guardians to participate in the questionnaire

“Questionnaire of the view of parents/guardians on managing fever in children”

Dear parents / guardians,

We are very grateful for your participation in this questionnaire. The purpose of this questionnaire is to clarify your practices and beliefs concerning febrile illness in your child. By gathering your answers and those of other participants, we aim to identify the ways how parental opinion could be taken into consideration to a greater extent when providing care for children with fever and their parents in future. The completion of the questionnaire will not take longer than 15 minutes.

The survey will include questions regarding the ongoing episode of your child’s illness, as well as questions on your general beliefs about fever in children.

Your consent or refusal to participate in this questionnaire will affect neither the management of your child’s illness in the hospital nor the attitude of the healthcare personnel towards you or your child. You have the right to refuse further participation at any moment, as well as to demand the withdrawal of data already given from being analysed for the study. In that case, please inform any of the healthcare personnel, or the research team about your decision.

Please, sign this consent for only if you intend that you and your child are enrolled in the study, and if you have been given sufficient information as described below.

Please mark each box with an „X” if you agree with the given statement.

1.	I confirm that I have read and understood the information provided in the informed consent form, and that I have been given a copy of it.	☐
2.	I confirm that I was given the opportunity to ask questions, that I received answers to my questions, and that I was given enough time to decide for or against participation in the study.	☐
3.	I confirm that I voluntarily agree to participate in this study, and that I am aware that I can withdraw my consent at any moment, without providing explanation. I understand that, by signing this form, I am not giving up any of my or my child’s constitutional rights.	☐
4.	I agree that my or my child’s personal information can be archived in a coded form, in accordance to requirements of data protection regulations, If I and my child are enrolled in a clinical study.	☐
5.	I confirm that I was not under any kind of pressure to participate in this study, and my consent to participate is voluntary.	☐

If you agree to participate in the study, please sign below.

(child's signature (if applicable)) /name, surname/

(signature of the parent or guardian) /name, surname/

(signature of the member of research team) /name, surname/

----

date

Note. Two signed copies of informed consent must be provided. One must be kept wih the participant, and the other must be given to the researcher.

The name of my child is:

In case of emergency, please contact

Name of the person:

Telephone number:
