# Supplementary material for: Beliefs, Practices and Health Care Seeking Behavior of Parents Regarding Fever in Children
Source: Medicina (Kaunas). 2019 Jul 22;55(7):398. doi: 10.3390/medicina55070398 (PMC6681325; doi:10.3390/medicina55070398)
Supplement: Supplementary file 1 [file medicina-55-00398-s001.zip › supplementary_files/Supplementary_file_1.pdf]

## Supplementary file No. 1: Questionnaire of the view of parents/guardians on managing fever in children (English version)

**Participant of the survey (circle the appropriate)** Mother, Father, Other________________

**No. of children in the family / household:** _________________

**Order of birth (**first / second/ third**) of the child that is our patient** ___________

**Age of mother (guardian)**: ______________

**Level of education of the mother (guardian) (circle the appropriate)**

1) Middle school (basic education)
2) High school
3) Professional ______________________
4) Unfinished higher education
5) Higher education (degree): ___________________________
6) Other______________________

**Age of father (guardian)**: ______________

**Level of education of the father (guardian) (circle the appropriate)**

1) Middle school (basic education)
2) High school
3) Professional ______________________
4) Unfinished higher education
5) Higher education (degree): ___________________________
6) Other______________________

1. **What body temperature in a child is a high fever, in your opinion?** _______________ °**C**
2. **At what temperature would you give your child medication to reduce it?** Abve............. °**C**
3. **What medication yould you give your child to reduce fever?**

   Ibuprofen (Nurofen, Ibustar, Ibumetin, Ibufen) ☐

   Paracetamol (Panadol, Efferalgan, calpol) ☐

   Other (which one........................ ) ☐
4. **How would you choose the dosage of medication?**
   1) As the doctor recommended ☐
   2) As the packaging says ☐
   3) Whatever I feel like, depends on the temperature ☐
   4) Other ☐
5. **In your opinion, can the child's body temperature during febrile illness increase to the a level that is dangerous to the child's life?**
   1) Yes ( Above.......... °**C**) ☐
   2) No ☐
   3) I don't know ☐

6. **Does fever itself indicate that the illness is serious?**
    1) yes ☐
    2) No ☐
    3) Other symptoms should be considered as well ☐
    4) I don't know ☐

7. **How soon after any of your children develops fever would you seek for medical help?**
    1) 0-6 hours ☐
    2) 6-12 hours ☐
    3) 12-24 hours ☐
    4) 24-48 hours (2nd day) ☐
    5) 48-72 hours (3rd day) ☐
    6) Later (when) _______________ ☐

8. **For how long had your child been ill THIS TIME before you sought medical help for the first time?**
    1) 0-6 hours ☐
    2) 6-12 hours ☐
    3) 12-24 hours ☐
    4) 24-48 hours (2 days) ☐
    5) 48-72 hours (3 days) ☐
    6) For loger _______________ ☐

9. **Day of the week (***for example, Sunday***) , when your child got ill: ____________________**

10. **Day of the week and time when you first sought help for your child (day, hh:mm)**

    __________________

11. **Where did you seek help first during this episode of the child's illness?**
    1) Family doctor ☐
    2) Out-of-hours telephone service ☐
    3) Out-of-hours primary care doctor ☐
    4) Ambulance ☐
    5) Hospital ☐
    6) Other ______________________________ ☐

**12. Was the explanation on the nature of illness and reasons for fever provided by the healthcare professiona mentioned above satisfactory?**

1) Yes ☐
2) No ☐
3) Partially ☐

**13. How did the information provided by the healthcare professional mentioned above affect your level of concern about the illness of your child?**

1) My level of concern was reduced ☐
2) My level of concern did not change ☐
3) My level of concern increased ☐

**14. Was the explanation on the nature of illness and reasons for fever provided by the doctor at the hospital (emergency department) satisfactory?**

1) Yes ☐
2) No ☐
3) Partially ☐

**15. How did the information provided by the doctor at the hospital (emergency department) affect your level of concern about the illness of your child?**

1) My level of concern was reduced ☐
2) My level of concern did not change ☐
3) My level of concern increased ☐

**16. How would you evaluate the availability of your family doctor?**

1) Very good ☐
2) Good ☐
3) More good than bad ☐
4) Normal ☐
5) More bad than good ☐
6) bad ☐
7) very bad ☐

17. **Does being treated in the hospital setting give you a better feeling of safety than care at home under supervison of your family doctor?**
    1) Yes ☐
    2) No ☐
    3) Partially ☐
